# C-reactive protein levels could be a prognosis predictor of prostate cancer: A meta-analysis

**DOI:** 10.3389/fendo.2023.1111277

**Published:** 2023-02-03

**Authors:** Kechong Zhou, Chao Li, Tao Chen, Xuejun Zhang, Baoluo Ma

**Affiliations:** ^1^ Department of Urology, Xiangyang Central Hospital, Affiliated Hospital of Hubei University of Arts and Science, Xiangyang, Hubei, China; ^2^ Department of Orthopedics, Xiangyang Central Hospital, Affiliated Hospital of Hubei University of Arts and Science, Xiangyang, Hubei, China

**Keywords:** meta-analysis, C-reactive protein, prostate cancer, prognosis, survival, crp

## Abstract

**Background:**

The relationship between the C-reactive protein (CRP) and prognosis in prostate cancer (PCa) has been widely discussed over the past few years but remains controversial.

**Material and methods:**

In our meta-analysis, we searched 16 reliable studies in the PubMed, Embase, and Cochrane Library databases. Otherwise, we have successfully registered on the INPLASY. We also performed random- and fixed-effects models to evaluate the hazard ratio (HR) and 95% confidence interval (CI), respectively.

**Result:**

The result of our meta-analysis shows that elevated CRP levels were related to worse overall survival (OS) (HR = 1.752, 95% CI = 1.304–2.355, *p* = 0.000), cancer-specific survival (CSS) (HR =1.823, 95%CI = 1.19-2.793, P = 0.006), *p* = 0.026), and progression-free survival (PFS) (HR = 1.663, 95% CI = 1.064–2.6, *p* = 0.026) of PCa patients. There was significant heterogeneity, so we performed a subgroup analysis according to the staging of the disease and found the same result. Furthermore, the heterogeneity was also reduced, and no statistical significance.

**Conclusion:**

Our study shows that the level of CRP could reflect the prognosis of prostate cancer patients. We find that PCa patients with high levels of CRP often have worse OS, CSS, and PFS, although the stages of the patients’ disease are different. More studies are needed to verify this idea.

## 1 Introduction

Prostate cancer (PCa) is one of the most common cancers, with a mortality rate that is among the top five worldwide. Moreover, it is the second most frequently diagnosed cancer among men (1). Most PCa patients are diagnosed when the disease is only localized, which means that many patients could be curable if the disease is detected in the early stages. In contrast, approximately 30% of patients will have cancer recurrence. Therefore, it is important to find more accurate prognoses and predictive markers for the treatment of PCa.

Before that, many indicators have been shown to be closely related to the prognosis of prostate cancer. For example, Gleason score, metastases, pain phosphatase, alkaline, and albumin were prognostic factors for several survival indicators of PCa (2–5). Moreover, systemic inflammation was discussed as a predictive factor for the survival of PCa patients in some studies (6). Some studies also showed that anti-inflammatory drugs had a protective effect on PCa patients (7).

C-reactive protein (CRP) is mainly produced by the liver as a typical acute-phase protein, which is one of the most common markers of systemic inflammation and is routinely measured (8). The elevation of CRP level was discussed as a prognostic indicator for many cancers, such as lung cancer, breast cancer, and colorectal cancer (9). It is also associated with the prognosis of urological cancers such as renal cell carcinoma (10, 11).

In the past few years, the relationship between CRP levels and the prognosis of prostate cancer patients remains controversial. Some studies suggested that PCa patients with elevated CRP levels often had worse survival (12, 13). Some other studies had different views and believed that there was no significant correlation between the CRP level and the prognosis of prostate cancer patients (14–16). The results of these studies were different, and due to their small sample size, the results were not very reliable. Therefore, we performed this meta-analysis by summarizing all credible articles to explore the relationship between C-reactive protein levels and prognosis in prostate cancer. Then, subgroup analysis was performed by staging the disease.

## 2 Materials and methods

### 2.1 Search strategy

We independently and systematically searched the Embase, PubMed, and Cochrane Library databases, and assessed the relationship between C-reactive protein levels and survival of prostate cancer patients up to October 2019. We used the following search terms: “Prostate Cancer”, “PCa”, “CRP”, and “C reactive protein”. Otherwise, we have successfully registered on the INPLASY, and our registration number was “INPALSY202060061”. There was no restriction on the type of study or the sample size.

### 2.2 Inclusion and exclusion criteria

Those who met the following conditions were eligible for inclusion in this study: 1) the studies discussed the relationship between CRP and the prognosis in PCa patients; 2) the study design was prospective, randomized controlled trials (RCTs) or retrospective studies; 3) contain data on hazard ratio (HR) and 95% confidence interval (CI) or include the survival curves of CRP in PCa patients. Studies were excluded based on the following criteria: 1) data cannot be obtained even after contacting the author; 2) the type of study was abstract, review, and comment; 3) duplicate studies.

### 2.3 Data abstraction

We gathered the following information for inclusion in the study by carefully reading and sifting through the retrieved titles and abstracts: 1) the basic characteristics of the study including the family name of the lead author, time of publication, nationality of patients, size of the sample, age, and staging of the disease; 2) time of follow-up, CRP cutoff values, and median follow-up; 3) HR, *p*-value, and 95% CIs of elevated CRP for all prognostic indicators, such as overall survival (OS), disease-specific survival (DSS), cancer-specific survival (CSS), recurrence-free survival (RFS), progression-free survival (PFS), and disease-free survival (DFS). It was worth mentioning that we have combined some similar prognostic indicators to make the study feasible. For example, CSS and DSS were considered to be CSS; PFS, RFS, and DFS were considered to be PFS. In order to ensure the effectiveness of the results, all results we have extracted were from multivariate analysis. When there was no exact HR reported, we extracted the data from its survival curve and calculated HR. We used the Newcastle–Ottawa Scale (NOS) to evaluate the quality of all included articles (17), and we also evaluated the selection, exposure, and comparability of these studies.

### 2.4 Statistical analysis

All statistical analyses were performed using Stata 12.0 software. Egger’s test and Begg’s funnel plot were used to assess the publication bias (18). The HR and 95% CIs were calculated to evaluate the correlation between C-reactive protein levels and the survival of PCa patients. We checked the heterogeneity among the included studies by using the chi-square test, and when *p* < 0.05, we considered the result significant. The higher the value of *I* ^2^, the higher the heterogeneity. We recognized that there was no significant heterogeneity when *I* ^2^ < 50%. The fixed-effects model (Mantel–Haenszel method) was adopted when no significant heterogeneity was detected (*p* > 0.05 and *I* ^2^ < 50%) (19). Otherwise, the random-effects model (DerSimonian and Laird method) was used (20). We also performed subgroup analyses when patients had different stages.

## 3 Results

### 3.1 Data retrieval

**Figure 1:**
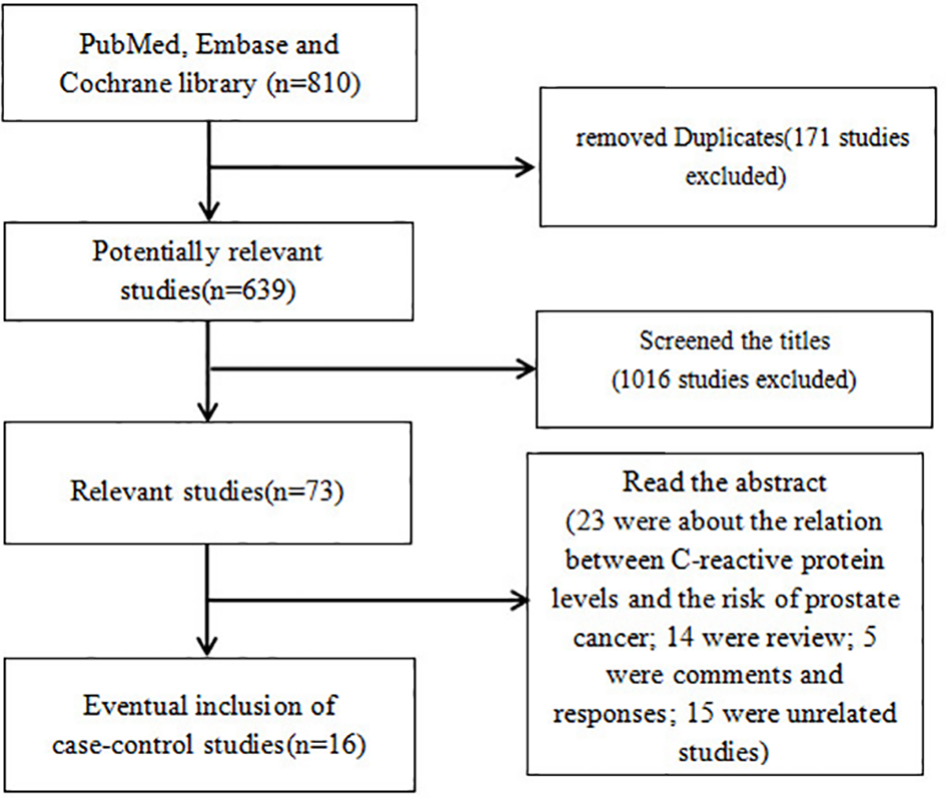
Flowchart illustrating the search strategy for CRP and prognosis in prostate cancer. CRP, C-reactive protein.

A total of 639 potentially relevant studies were identified from the aforementioned databases. As shown in **Figure 1**, the full text of 73 studies on the association between C-reactive protein levels and prognosis in prostate cancer was retrieved after screening the titles. A total of 57 studies were excluded after screening the abstract: 23 were about the association between C-reactive protein levels and the risk of prostate cancer; 14 were reviews; 5 were comments and responses; 15 were unrelated studies. Finally, 16 studies were pooled in the final meta-analysis, which contained 13,555 PCa patients.

### 3.2 Study characteristics

The main features of all included studies are presented in **Table 1**. The patients were from the United Kingdom, Russia, Japan, Canada, Austria, Sweden, China, and the United States. The research population was also different: five were metastatic prostate cancer (mPCa) patients, four were localized PCa patients, and the remaining were not clearly stated. Otherwise, CRP values were analyzed by different techniques in each study that we included. A total of 12 studies used a dichotomous variable to analyze CRP with different cutoff values. Only one study dealt with trichotomous variables and compared the survival between the highest tertile and the lowest tertile. CRP was considered a continuous variable in the remaining three studies, in which HR was calculated as a unit change on a log scale. All studies showed the values of HR and confidence interval (**Table 2**). Significantly, all data with multivariate analysis were collected to ensure the credibility of the results.

**Table 1:** Main characters of studies included in this meta-analysis.

First author	Year	Country	Sample size	Age	Treatment	Cutoff (mg/L)	Median follow-up
McArdle PA	2006	UK	62	NR	NR	10	62 m
Beer TM	2008	USA and Canada	160	68.0 (45–92)	Endocrine	8	NR
Nakashima J	2008	Japan	126	NR	NR	1.5	39.7 m (1–144)
Stark JR	2009	USA	601	68.6	NR	1.7	NR
McArdle PA	2010	UK	98	NR	NR	3.10	10 y
McArdle PA	2010	UK	98	NR	NR	3.10	10 y
Ito M	2011	Japan	80	NR	Docetaxel	5	9.4 m (1–31)
McCall P	2012	UK	61	70 (63–75)	NR	IHC	8.4 y (5.7–11)
Pond GR	2012	Russia and USA	110	NR	Docetaxel–prednisone	Log	18
Pond GR	2012	Russia and USA	110	NR	Docetaxel–prednisone	Log	18
Prins RC	2012	USA	119	71.9 (45.8–91.5)	NR	Log	19.7 m (0.9–98.5)
Hall WA	2013	USA	54	45–74	RP	Log	NR
Hall WA	2013	USA	152	43–83	RT	Log	NR
Matsuyama H	2014	Japan	279	71 (48–91)	Docetaxel	3.2	94 m (81–101)
Thurner E	2015	Austria	261	67.9	NR	8.6	80 m (76.3–83.7)
Thurner E	2015	Austria	261	67.9	NR	8.6	80 m (76.3–83.7)
Thurner E	2015	Austria	261	67.9	NR	8.6	80 m (76.3–83.7)
Xu LY	2015	China	135	NR	NR	10	NR
Liao SG	2016	China	115	74.8	NR	8	NR
Liao SG	2016	China	115	74.8	NR	8	NR
Sevcenco S	2016	European and American	7,205	61 (57–66)	NR	5	27 m (19–48)
Aryhur R	2018	Swedish	779	NR	NR	10	NR
Aryhur R	2018	Swedish	1,741	NR	NR	10	NR

RP, radical prostatectomy; RT, radiotherapy; IHC, immunohistochemistry; m, months; y, years; NR, not reported.

**Table 2:** Main data of studies included in this meta-analysis.

First author	Year	Disease	Survival analysis	Multivariate analysis	*p*
				HR (95% CI)	
McArdle PA	2006	mPCa	CSS	1.97 (0.99–3.92)	0.052
Beer TM	2008	mAIPCa	OS	1.405 (1.199–1.647)	<0.0001
Nakashima J	2008	mPCa	DSS	1.884 (1.028–3.454)	0.0404
Stark JR	2009	PCa	CSS	1.48 (0.83–2.66)	0.08
McArdle PA	2010	Localized PCa	OS	1.60 (1.03–2.47)	0.036
McArdle PA	2010	Localized PCa	CSS	1.88 (1.01–3.52)	0.048
Ito M	2011	CRPCa	OS	1.95 (1.33–2.96)	<0.001
McCall P	2012	Hormone-naive advanced prostate cancer	DSS	4.3 (1.5–12.4)	0.009
Pond GR	2012	mCRPCa	OS	1.38 (1.12–1.70)	0.003
Pond GR	2012	mCRPCa	PFS	1.44 (1.17–1.77)	< 0.001
Prins RC	2012	CRPCa	OS	1.106 (1.022–1.197)	0.013
Hall WA	2013	Localized PCa	RFS	1.48 (0.68–3.21)	0.325
Hall WA	2013	Localized PCa	RFS	2.03 (1.19–3.47)	0.009
Matsuyama H	2014	CRPCa	OS	1.94 (1.08–3.55)	0.0268
Thurner E	2015	Localized PCa	OS	3.24 (1.84–5.71)	<0.001
Thurner E	2015	Localized PCa	CSS	4.31 (1.22–15.1)	0.023
Thurner E	2015	Localized PCa	DFS	2.07 (1.02–4.17)	0.043
Xu LY	2015	mPCa	OS	2.39 (1.56–3.69)	<0.001
Liao SG	2016	CRPCa	OS	2.003 (1.285–3.121)	0.002
Liao SG	2016	CRPCa	PFS	2.184 (1.401–3.403)	0.001
Sevcenco S	2016	Localized PCa	RFS	1.23 (1.04–1.45)	NR
Aryhur R	2018	PCa	CSS	1.00 (0.92–1.10)	NR
Aryhur R	2018	PCa	OS	0.97 (0.89–1.06)	NR

HR, hazard ratio; CI, credibility interval; mPCa, metastatic prostate cancer; mAIPCa, metastatic androgen-independent prostate cancer; PCa, prostate cancer; CRPCa, castration‑resistant prostate cancer; OS, overall survival; CSS, cancer-specific survival; DSS, disease-specific survival; PFS, progression free survival; RFS, recurrence-free survival; DFS, disease free survival; NR, not report.

### 3.3 Meta-analysis

**Figure 2:**
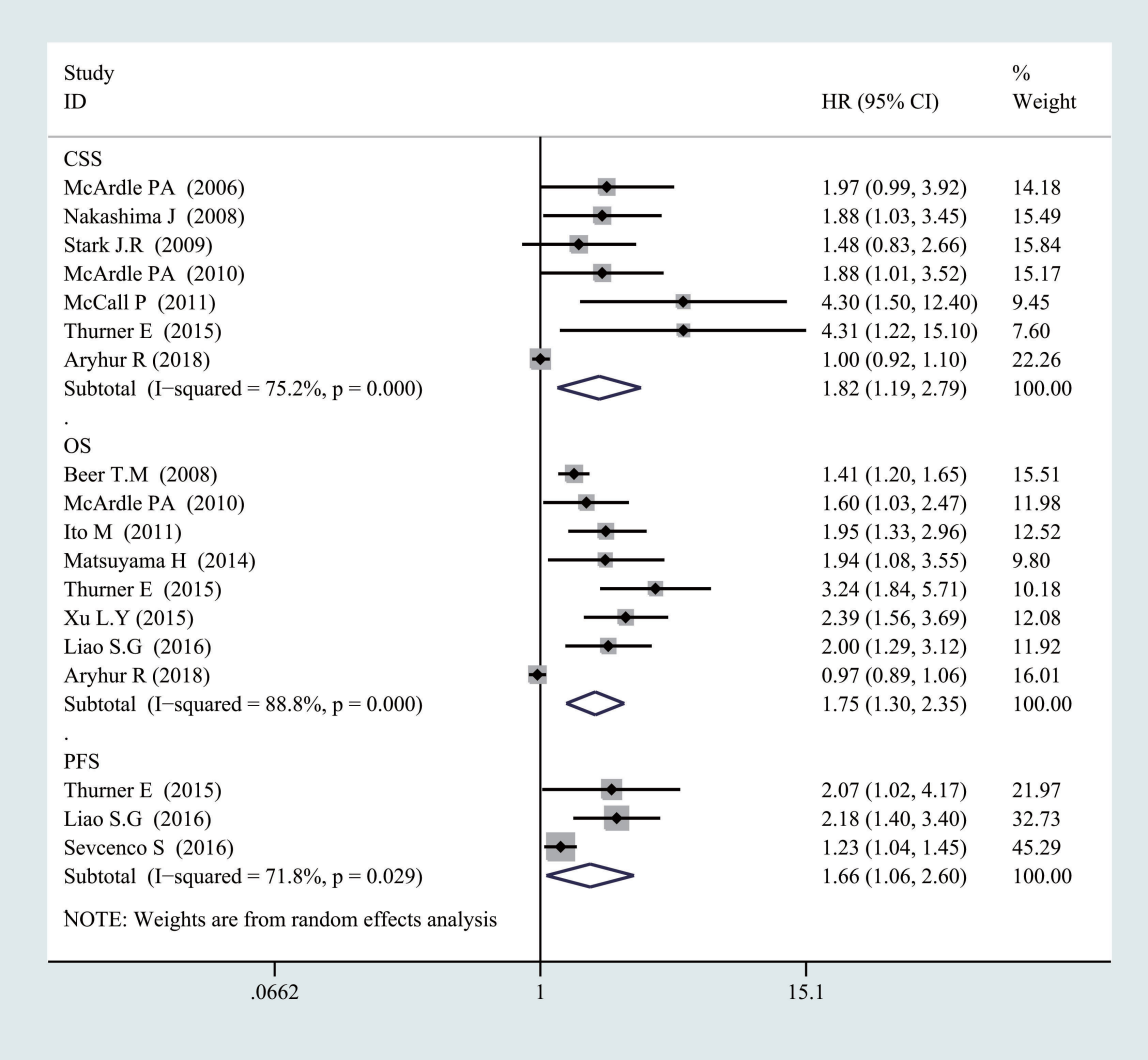
Forest plot of categorized CRP and prognosis in prostate cancer from random-effects analysis. CRP, C-reactive protein.

**Figure 3:**
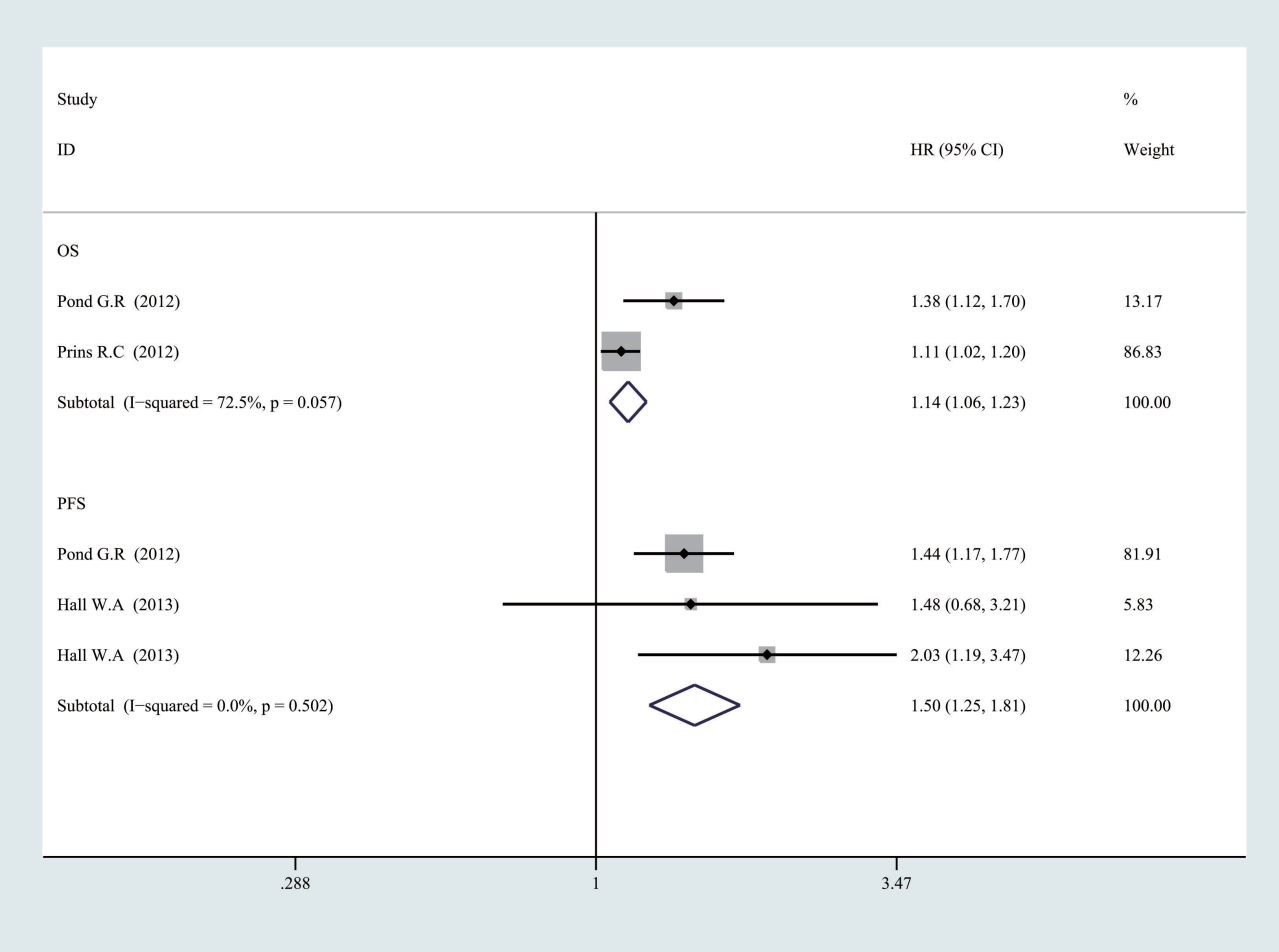
Forest plot of log CRP and prognosis in prostate cancer from fixed-effects analysis. CRP, C-reactive protein.

By analyzing all studies, we found that there was a significant correlation between C-reactive protein levels and prognosis in PCa patients (**Figures 2**, **3**). According to the high heterogeneity (*I* ^2^ > 50%), the random-effects model was carried out to calculate the pooled HR and their 95% CI. Otherwise, the subgroup analyses were also performed as follows to reduce heterogeneity.

#### 3.3.1 Overall survival

For OS, there was significant heterogeneity between studies of categorized CRP (*I* ^2^ = 0.888, *p* = 0.000), and log CRP (*I* ^2^ = 0.725, *p* = 0.057) has no significant heterogeneity. Elevated serum CRP level was significantly associated with the OS of PCa for categorized data (HR = 1.752, 95% CI = 1.304–2.355, *p* = 0.000) and log CRP (HR = 1.142, 95% CI = 1.059–1.232, *p* = 0.001) (**Figures 2**, **3**).

#### 3.3.2 Cancer-specific survival

For CSS, seven studies discussed the association between the high CRP level and worse CSS for categorized CRP, and the pooled HR was 1.823 (95% CI = 1.19–2.793, *p* = 0.006). There was also significant heterogeneity (*I* ^2^ = 0.752, *p* = 0.000). Only one study discussed log CRP (**Figures 2**, **3**).

#### 3.3.3 Progression-free survival

For PFS, there was also a significant association between the high CRP level and worse PFS for categorized CRP (HR = 1.663, 95% CI = 1.064–2.6, *p* = 0.026) and log CRP (HR = 1.504, 95% CI = 1.247–1.814, *p* = 0.000). Some heterogeneity was also found for categorized CRP (*I* ^2^ = 0.718, *p* = 0.029) (**Figures 2**, **3**).

In summary, a high CRP level was proved to be associated with a worse OS, CSS, and PFS, which meant that serum CRP level could be a prognostic biomarker for the survival of PCa patients. However, there was also significant heterogeneity between the studies that we included. Therefore, we performed a subgroup analysis by the staging of the disease to find the sources of heterogeneity.

#### 3.3.4 Relationship between high CRP level and prognosis in mPCa

**Figure 4:**
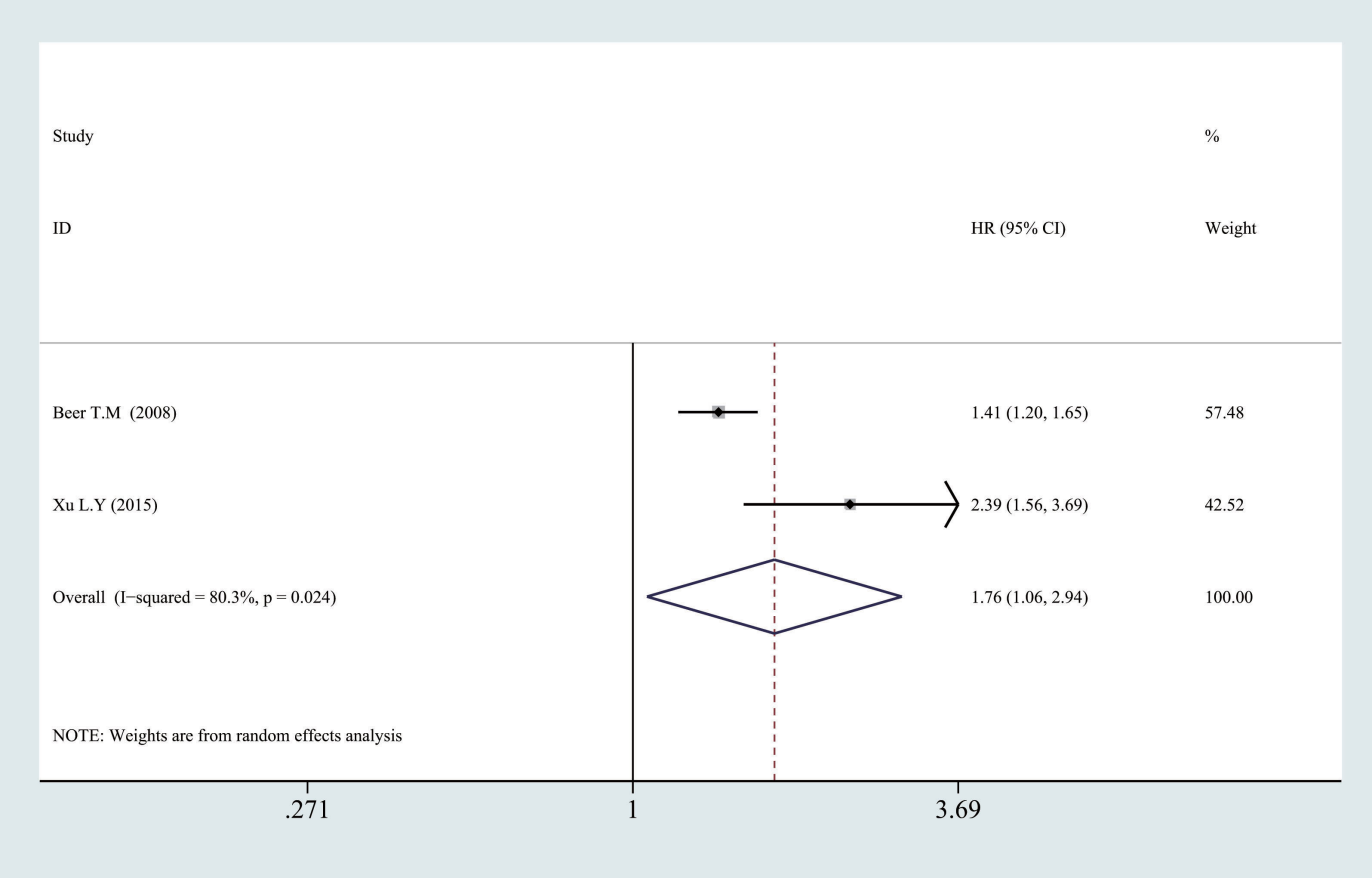
Relationship between categorized CRP and prognosis in mPCa. CRP, C-reactive protein; mPCa, metastatic prostate cancer.

In the subgroup analysis, we also found a significant association between high CRP levels and worse OS in mPCa by pooled HR of two studies (HR = 1.765, 95% CI = 1.058–2.943, *p* = 0.029) (**Figure 4**).

#### 3.3.5 Relationship between high CRP level and prognosis in localized PCa

**Figure 5:**
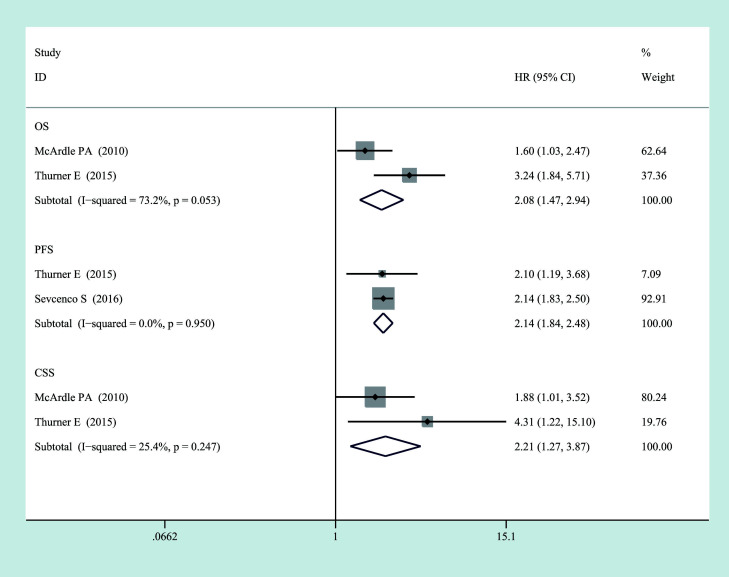
Relationship between categorized CRP and prognosis in localized PCa. CRP, C-reactive protein; PCa, prostate cancer.

As shown in **Figure 5**, a significant association also could be observed between elevated CRP levels and worse OS (HR = 2.083, 95% CI = 1.473–2.944, *p* = 0.000, *I* ^2^ = 0.732, *p* = 0.053), CSS (HR = 2.215, 95% CI = 1.266–3.874, *p* = 0.005, *I* ^2^ = 0.000, *p* = 0.95), and PFS (HR = 2.137, 95% CI = 1.839–2.484, *p* = 0.000, *I* ^2^ = 0.254, *p* = 0.247) in localized PCa for categorized CRP. Significantly, all heterogeneity between included studies was reduced, and there was no significant heterogeneity between studies on localized PCa (*p* > 0.05, *I* ^2^ < 50%). It indicated that the staging of the disease was likely to be the source of heterogeneity.

### 3.4 Publication bias

**Figure 6:**
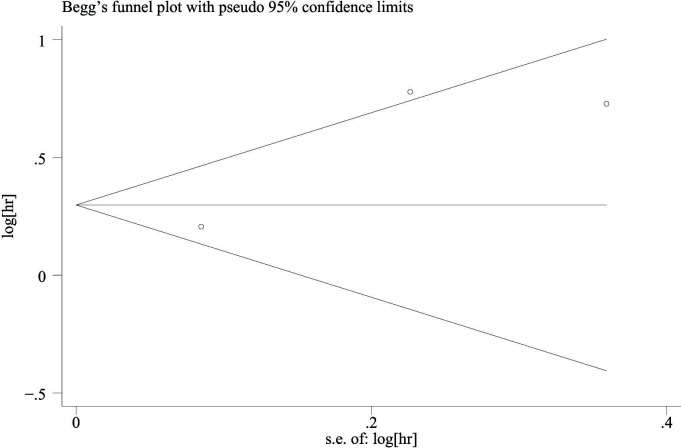
Funnel plot (categorized CRP and PFS in prostate cancer). CRP, C-reactive protein; PFS, progression-free survival.

As shown in **Figure 6**, we also used Egger’s test and Begg’s funnel plot to evaluate the publication bias. The results showed that there was no publication bias because of the symmetric shapes of all models (*p* = 0.252).

### 3.5 Sensitivity analysis

**Figure 7:**
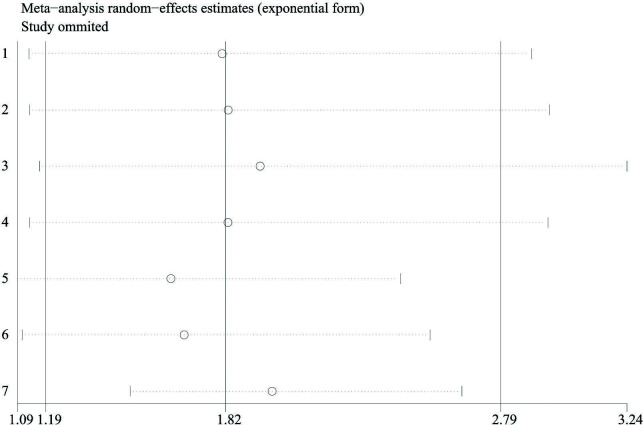
Sensitivity analysis (CSS in prostate cancer). CSS, cancer-specific survival.

Sensitivity analysis was used to assess one study’s effect on the total analytical results. **Figure 7** shows the sensitivity analysis of the subgroup of CSS. No study impacted the pooled HRs significantly, which meant that our results were reliable.

## 4 Discussion

This was a meta-analysis of C-reactive protein levels and prognosis in prostate cancer. We found that PCa patients often had worse survival with high levels of CRP. The results further confirmed the study of Liu et al. (21). Moreover, we also performed subgroup analyses by staging the disease because of the bigger sample size. Our study also indicated that the level of CRP was related to the survival of both mPCa patients and localized PCa patients.

As prostate cancer is the most common malignancy in the male reproductive system, its treatment is varied. It could be roughly divided into wait and watch, active monitoring, surgical treatment, radiotherapy, androgen deprivation therapy (ADT), etc. (22). However, mPCa is still deadly, with a 5-year survival rate of approximately 30%, indicating the need for better treatment options. In recent years, there are also some new advances in the treatment of prostate cancer. Arpit et al. found that rucaparib and olaparib (poly-ADP-ribose polymerase (PARP) inhibitors) could be used as the targeted therapy option for patients with mPCa (23). Arnas et al. suggested that high-intensity focused ultrasound (HIFU) has a high control rate and safety in the treatment of local prostate cancer patients and should be promoted in clinical treatment (24). Jiang et al. also found that nanotechnology could create good synergy with radiotherapy, chemotherapy, thermotherapy, photodynamic therapy, and gene therapy, which could increase the effectiveness of treatment and reduce drug resistance (25). With the recent advances, the treatments of prostate cancer became varied, so it was particularly important to evaluate the prognosis of prostate cancer patients.

In recent years, diversified predictors have been confirmed for predicting the prognosis of PCa. One of the most commonly used prostate-specific antigens was the conventional measurement index in the treatment of all PCa patients. In addition, many studies found that epidermal growth factor receptor (EGFR), pAkt, nuclear factor-kappa B, macrophage inhibitory cytokine-1 (MIC-1), matrix metalloproteinase-1 (MMP-1), MMP-9 and tissue inhibitor of metalloproteinase-2 (TIMP-2), and macrophage inhibitory cytokine-1 were associated with the outcome of PCa patients (26, 27). However, in fact, all the above biomarkers must be tested in pathological tissues. This made it difficult for real-time monitoring of the prognosis of patients. Instead, the inflammation indicators were easy to examine from the blood. Brown et al. found that an inflammation-based prognostic score (Glasgow Prognostic Score) was associated with advanced lung and gastrointestinal cancers for the first time (28). After that, the Glasgow Prognostic Score was found to be related to many types of cancer. Recently, one study found that the prognostic value of the Glasgow Prognostic Score in PCa patients, the elevation of CRP (>10 mg/L), and hypoalbuminemia (<35 g/L) were related to worse prognoses of PCa patients (6). William Khalil et al. also summarized the hematological indicators related to the prognosis of prostate cancer, including collagenases, stromelysins, TIMP-1 and TIMP-2, MMP-13, osteopontin (OPN), MMP-2, MMP-9, and MMP-7 (29). Maria et al. also considered the following blood indicators to be associated with the prognosis of prostate cancer: prostate-specific antigen (PSA) + kallikrein antigen (KLK2), AKT, chromogranin A (CHGA), and early prostate cancer antigen (EPCA) (30). Katalin et al. also considered that the following blood-derived biomarkers played an important role in the prognosis of prostate cancer: circulating tumor cells, cellular and soluble immunological and inflammation-related blood markers, and extracellular vesicles and their microRNA content (31). However, the inflammation indicators could be more easily detected from the blood and are widely used in the clinical setting. CRP is the most common indicator of inflammation and has also been widely studied in recent years.

There are many possible mechanisms for the elevation of CRP with worse survival in cancer patients. Chronic inflammation could promote the growth of vascular endothelial cells, which was beneficial to the occurrence of tumors (32). On the one hand, the inflammatory reaction could be activated by the rapid growth of the tumor. Many inflammatory factors are released when the tumor is growing. On the other hand, inflammation also could provide a microenvironment for the growth of tumors, for example, survival factors and growth factors (33). Moreover, a significant negative correlation was recently found between the elevation of CRP and T-lymphocyte subset infiltration (34). Therefore, our study is necessary for the advancement in the treatment of PCa.

The level of CRP is also important for the outcome in PCa. Sevcenco et al. suggested that patients are more prone to experience biochemical recurrence (BCR) with high levels of CRP (35). Hall et al. performed multivariable analysis and found that a higher CRP level is an independent prognostic factor for BCR in patients with radiotherapy (36). According to the multivariate analysis of McArdle et al., a CRP level >10 mg dl^−1^ before a diagnosis is an independent prognostic factor for both OS and CSS of PCa patients (37). Otherwise, Arthur et al. found that elevated CRP is associated with increased odds of both a high risk and metastatic PCa and high PSA levels, which means that the level of CRP would rise with the increase of tumor stage and disease progression for PCa patients (38). Sevcenco et al. also found that the patients with higher Gleason scores on biopsy, lymph node metastasis, seminal vesicle invasion, extracapsular extension, and positive surgical margins status often had a higher level of CRP compared with patients without these features (35). This further confirmed this conclusion, but more studies are needed to confirm the conclusions.

Firstly, we analyzed the association between the elevation of CRP and the prognosis of PCa patients. We found that CRP level could be a prognosis predictor of PCa in both categorized data and log data. The result showed that elevated CRP levels were associated with worse OS, CSS, and PFS in PCa patients. However, significant heterogeneity was observed; the heterogeneity might be due to many aspects, for example, basic characteristics, the staging of the disease, follow-up time, the difference in treatment, and the different cutoff values. Therefore, we chose a random-effects model to reduce the effect of these differences. Otherwise, we performed a subgroup analysis by the staging of the disease according to the characteristics of these studies and found that the heterogeneity was significantly reduced, which meant that the staging of the disease was likely to be the source of heterogeneity. The result further confirmed that CRP level could be a prognosis predictor for PCa.

For mPCa, we found a significant association between high CRP levels and worse OS in mPCa. For localized PCa, a significant association also could be observed between elevated CRP levels and worse OS, CSS, and PFS. Furthermore, we found that heterogeneity was significantly reduced, which indicated that the staging of the disease contributed to the source of heterogeneity. Our meta-analysis proved that an elevated CRP level is a strong prognosis predictor of PCa patients, which could be helpful for the treatment of PCa. It also could help doctors better monitor the progress of the disease.

Our research includes more new studies compared with the research of Liu et al. (21). Moreover, we also performed Begg’s test to make our results more credible and the subgroup analysis by staging the disease, explored the relationship between CRP and the survival of patients with different stages of PCa, and found the source of heterogeneity.

However, our study also has some limitations. There is also a need for more credible studies to confirm our conclusions, although we have reviewed all the current literature. The heterogeneity was significant in our study, but we performed stratified analysis to reduce the heterogeneity, which made the results more credible. In addition, the techniques for detecting CRP were different, which made the results unreliable.

## 5 Conclusion

In general, our meta-analysis found that elevated CRP levels were related to worse OS, CSS, and PFS in PCa patients. Furthermore, we also observed the same relationship between CRP and the survival of localized PCa patients. Therefore, it can be indicated that the CRP level could be used as a prognosis predictor of prostate cancer. More studies are needed to confirm these conclusions.

## Data availability statement

The original contributions presented in the study are included in the article/supplementary material. Further inquiries can be directed to the corresponding author.

## Author contributions

i) Conception and design: KZ. ii) Administrative support: BM. iii) Provision of study materials or patients: KZ and XZ. iv) Collection and assembly of data: BM, CL and KZ. v) Data analysis and interpretation: KZ, CL and TC. vi) Manuscript writing: KZ and CL. All authors contributed to the article and approved the submitted version.

## References

[1] Torre LA Bray F Siegel RL Ferlay J Lortet-Tieulent J Jemal A . Global cancer statistics, 2012. CA: A Cancer J Clin (2015) 65:87. doi: 10.3322/caac.21262 25651787

[2] Halabi S Vogelzang NJ Kornblith AB Ou SS Kantoff PW Dawson NA . Pain predicts overall survival in men with metastatic castration-refractory prostate cancer. J Clin Oncol (2008) 26:2544. doi: 10.1200/JCO.2007.15.0367 18487572

[3] Armstrong AJ Garrett-Mayer ES Yang YC de Wit R Tannock IF Eisenberger M . A contemporary prognostic nomogram for men with hormone-refractory metastatic prostate cancer: A TAX327 study analysis. Clin Cancer Res (2007) 13:6396. doi: 10.1158/1078-0432.CCR-07-1036 17975152

[4] Halabi S Small EJ Kantoff PW Kattan MW Kaplan EB Dawson NA . Prognostic model for predicting survival in men with hormone-refractory metastatic prostate cancer. J Clin Oncol (2003) 21:1232. doi: 10.1200/JCO.2003.06.100 12663709

[5] Smaletz O Scher HI Small EJ Verbel DA McMillan A Regan K . Nomogram for overall survival of patients with progressive metastatic prostate cancer after castration. J Clin Oncol (2002) 20:3972. doi: 10.1200/JCO.2002.11.021 12351594

[6] Shafique K Proctor MJ McMillan DC Qureshi K Leung H Morrison DS . Systemic inflammation and survival of patients with prostate cancer: Evidence from the Glasgow inflammation outcome study. Prostate Cancer Prostatic Dis (2012) 15:195. doi: 10.1038/pcan.2011.60 22343838

[7] Mahmud S Franco E Aprikian A . Prostate cancer and use of nonsteroidal anti-inflammatory drugs: Systematic review and meta-analysis. Br J Cancer (2004) 90:93. doi: 10.1038/sj.bjc.6601416 14710213 PMC2395299

[8] Hurlimann J Thorbecke GJ Hochwald GM . The liver as the site of c-reactive protein formation. J Exp Med (1966) 123:365. doi: 10.1084/jem.123.2.365 4379352 PMC2138142

[9] Roxburgh CS McMillan DC . Role of systemic inflammatory response in predicting survival in patients with primary operable cancer. Future Oncol (2010) 6:149. doi: 10.2217/fon.09.136 20021215

[10] Saito K Kihara K . Role of c-reactive protein in urological cancers: A useful biomarker for predicting outcomes. Int J UROL (2013) 20:161. doi: 10.1111/j.1442-2042.2012.03121.x 22897628

[11] Wu Y Fu X Zhu X He X Zou C Han Y . Prognostic role of systemic inflammatory response in renal cell carcinoma: A systematic review and meta-analysis. J Cancer Res Clin Oncol (2011) 137:887. doi: 10.1007/s00432-010-0951-3 20878529 PMC11828339

[12] Beer TM Lalani AS Lee S Mori M Eilers KM Curd JG . C-reactive protein as a prognostic marker for men with androgen-independent prostate cancer. CANCER-AM Cancer Soc (2008) 112:2377. doi: 10.1002/cncr.23461 18428198

[13] Thurner E Krenn-Pilko S Langsenlehner U Stojakovic T Pichler M Gerger A . The elevated c-reactive protein level is associated with poor prognosis in prostate cancer patients treated with radiotherapy. Eur J Cancer (2015) 51:610. doi: 10.1016/j.ejca.2015.01.002 25618827

[14] McArdle PA Mir K Almushatat ASK Wallace AM Underwood MA McMillan DC . Systemic inflammatory response, prostate-specific antigen and survival in patients with metastatic prostate cancer. UROL Int (2006) 77:127. doi: 10.1159/000093905 16888416

[15] Stark JR Li H Kraft P Kurth T Giovannucci EL Stampfer MJ . Circulating prediagnostic interleukin-6 and c-reactive protein and prostate cancer incidence and mortality. Int J Cancer (2009) 124:2683. doi: 10.1002/ijc.24241 19189403 PMC2667697

[16] Elsberger B Lankston L McMillan DC Underwood MA Edwards J . Presence of tumoural c-reactive protein correlates with progressive prostate cancer. Prostate Cancer Prostatic Dis (2011) 14:122. doi: 10.1038/pcan.2011.5 21358753

[17] Stang A . Critical evaluation of the Newcastle-Ottawa scale for the assessment of the quality of nonrandomized studies in meta-analyses. Eur J Epidemiol (2010) 25:603. doi: 10.1007/s10654-010-9491-z 20652370

[18] Hayashino Y Noguchi Y Fukui T . Systematic evaluation and comparison of statistical tests for publication bias. J Epidemiol (2005) 15:235. doi: 10.2188/jea.15.235 16276033 PMC7904376

[19] DerSimonian R Laird N . Meta-analysis in clinical trials revisited. CONTEMP Clin TRIALS (2015) 45:139. doi: 10.1016/j.cct.2015.09.002 26343745 PMC4639420

[20] Mantel N Haenszel W . Statistical aspects of the analysis of data from retrospective studies of disease. J Natl Cancer Inst (1959) 22:719. 13655060

[21] Liu Z Chu L Fang J Zhang X Zhao H Chen Y . Prognostic role of c-reactive protein in prostate cancer: A systematic review and meta-analysis. Asian J ANDROL (2014) 16:467. doi: 10.4103/1008-682X.123686 24589465 PMC4023380

[22] Achard V Putora PM Omlin A Zilli T Fischer S . Metastatic prostate cancer: Treatment options. Oncology (2022) 100:48. doi: 10.1159/000519861 34781285

[23] Rao A Moka N Hamstra DA Ryan CJ . Co-Inhibition of androgen receptor and PARP as a novel treatment paradigm in prostate cancer-where are we now? Cancers (Basel) (2022) 14(3):801. doi: 10.3390/cancers14030801 PMC8834038 35159068

[24] Bakavicius A Marra G Macek P Robertson C Abreu AL George AK . Available evidence on HIFU for focal treatment of prostate cancer: A systematic review. Int Braz J UROL (2022) 48:263. doi: 10.1590/s1677-5538.ibju.2021.0091 34003610 PMC8932027

[25] Zhao J Zhang C Wang W Li C Mu X Hu K . Current progress of nanomedicine for prostate cancer diagnosis and treatment. BioMed Pharmacother (2022) 155:113714. doi: 10.1016/j.biopha.2022.113714 36150309

[26] Mimeault M Johansson SL Batra SK . Pathobiological implications of the expression of EGFR, pAkt, NF-kappaB and MIC-1 in prostate cancer stem cells and their progenies. PloS One (2012) 7:e31919. doi: 10.1371/journal.pone.0031919 22384099 PMC3285632

[27] Ozden F Saygin C Uzunaslan D Onal B Durak H Aki H . Expression of MMP-1, MMP-9 and TIMP-2 in prostate carcinoma and their influence on prognosis and survival. J Cancer Res Clin Oncol (2013) 139:1373. doi: 10.1007/s00432-013-1453-x 23708302 PMC11824703

[28] Brown DJF Milroy R Preston T McMillan DC . The relationship between an inflammation-based prognostic score (Glasgow prognostic score) and changes in serum biochemical variables in patients with advanced lung and gastrointestinal cancer. J Clin Pathol (2007) 60:705. doi: 10.1136/jcp.2005.033217 16644880 PMC1955069

[29] El-Chaer WK Moraes CF Nóbrega OT . Diagnosis and prognosis of prostate cancer from circulating matrix metalloproteinases and inhibitors. J Aging Res (2018) 2018:1. doi: 10.1155/2018/7681039 PMC6079523 30123587

[30] Adamaki M Zoumpourlis V . Prostate cancer biomarkers: From diagnosis to prognosis and precision-guided therapeutics. Pharmacol THERAPEUT (2021) 228:107932. doi: 10.1016/j.pharmthera.2021.107932 34174272

[31] Balázs K Antal L Sáfrány G Lumniczky K . Blood-derived biomarkers of diagnosis, prognosis and therapy response in prostate cancer patients. J Personalized Med (2021) 11:296. doi: 10.3390/jpm11040296 PMC8070149 33924671

[32] Xavier P Belo L Beires J Rebelo I Martinez-de-Oliveira J Lunet N . Serum levels of VEGF and TNF-α and their association with c-reactive protein in patients with endometriosis. Arch GYNECOL OBSTET (2006) 273:227. doi: 10.1007/s00404-005-0080-4 16208475

[33] Hanahan D Weinberg RA . Hallmarks of cancer: The next generation. CELL (2011) 144:646. doi: 10.1016/j.cell.2011.02.013 21376230

[34] Canna K McArdle PA McMillan DC McNicol AM Smith GW McKee RF . The relationship between tumour T-lymphocyte infiltration, the systemic inflammatory response and survival in patients undergoing curative resection for colorectal cancer. Br J Cancer (2005) 92:651. doi: 10.1038/sj.bjc.6602419 15700032 PMC2361875

[35] Sevcenco S Mathieu R Baltzer P Klatte T Fajkovic H Seitz C . The prognostic role of preoperative serum c-reactive protein in predicting the biochemical recurrence in patients treated with radical prostatectomy. Prostate Cancer Prostatic Dis (2016) 19:163. doi: 10.1038/pcan.2015.60 26810014

[36] Hall WA Nickleach DC Master VA Prabhu RS Rossi PJ Godette K . The association between c-reactive protein (CRP) level and biochemical failure-free survival in patients after radiation therapy for nonmetastatic adenocarcinoma of the prostate. CANCER-AM Cancer Soc (2013) 119:3272. doi: 10.1002/cncr.28185 23818401

[37] McArdle PA Qayyum T McMillan DC . Systemic inflammatory response and survival in patients with localised prostate cancer: 10-year follow-up. UROL Int (2010) 85:482. doi: 10.1159/000320242 21071914

[38] Arthur R Williams R Garmo H Holmberg L Stattin P Malmstrom H . Serum inflammatory markers in relation to prostate cancer severity and death in the Swedish AMORIS study. Int J Cancer (2018) 142:2254. doi: 10.1002/ijc.31256 29322512

