# Corrigendum: C-reactive protein levels could be a prognosis predictor of prostate cancer: A meta-analysis

**DOI:** 10.3389/fendo.2023.1176102

**Published:** 2023-03-30

**Authors:** Kechong Zhou, Chao Li, Tao Chen, Xuejun Zhang, Baoluo Ma

**Affiliations:** ^1^ Department of Urology, Xiangyang Central Hospital, Affiliated Hospital of Hubei University of Arts and Science, Xiangyang, Hubei, China; ^2^ Department of Orthopedics, Xiangyang Central Hospital, Affiliated Hospital of Hubei University of Arts and Science, Xiangyang, Hubei, China

**Keywords:** meta-analysis, C-reactive protein, prostate cancer, prognosis, survival, crp

## Text correction

In the published article, there was an error. There was a mistake in the Abstract. The result of cancer-specific survival (CSS) was displayed as “HR = 1.663, 95%CI = 1.064-2.6, P = 0.026”.

A correction has been made to the Abstract, Paragraph 1. This sentence previously stated:

“[HR = 1.663, 95%CI = 1.064-2.6, P = 0.026]”

The corrected sentence appears below:

“[HR =1.823, 95%CI = 1.19-2.793, P = 0.006]”

The authors apologize for this error and state that this does not change the scientific conclusions of the article in any way. The original article has been updated.

